# Electrically Tunable Optical Metasurfaces for Dynamic Polarization Conversion

**DOI:** 10.1021/acs.nanolett.1c02318

**Published:** 2021-07-21

**Authors:** Ping Yu, Jianxiong Li, Na Liu

**Affiliations:** † Max Planck Institute for Solid State Research, Heisenbergstrasse 1, 70569 Stuttgart, Germany; ‡ 2nd Physics Institute, University of Stuttgart, Pfaffenwaldring 57, 70569 Stuttgart, Germany

**Keywords:** metasurfaces, electrical control, polarization conversion, holography, visible frequencies

## Abstract

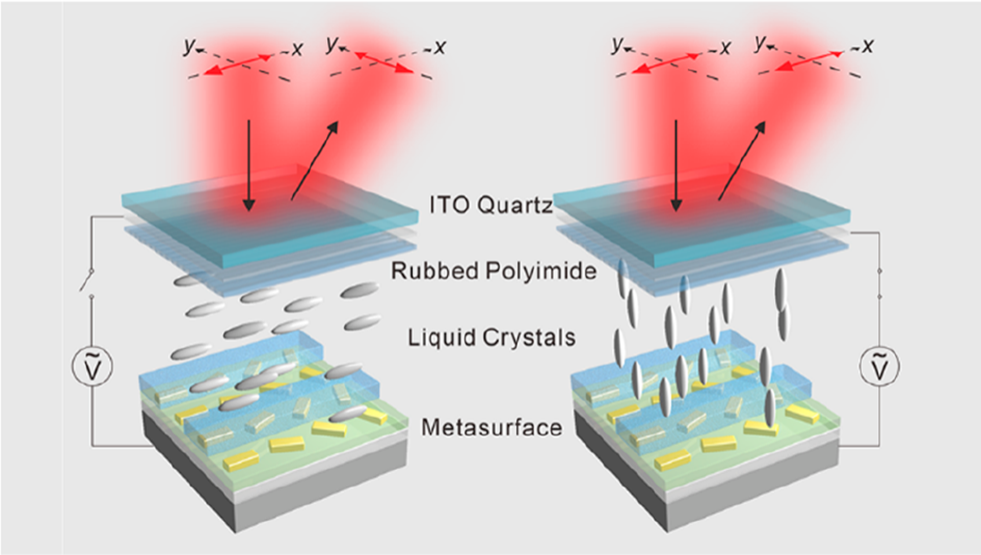

Dynamic control over the polarization of light is highly desirable in many optical applications, including optical communications, laser science, three-dimensional displays, among others. Conventional methods for polarization control are often based on bulky optical elements. To achieve highly integrated optical devices, metasurfaces, which have been intensively studied in recent years, hold great promises to replace conventional optical elements for a variety of optical functions. In this work, we demonstrate electrically tunable optical metasurfaces for dynamic polarization conversion at visible frequencies. By exploring both the geometric and propagation phase tuning capabilities, rapid and reversible polarization rotation up to 90° is achieved for linearly polarized light. The dynamic functionality is imparted by liquid crystals, which serve as a thin surrounding medium with electrically tunable refractive indices for the metasurface antennas. Furthermore, we expand our concept to demonstrate electrically tunable metasurfaces for dynamic holography and holographic information generation with independently controlled multiple pixels.

The manipulation of light polarization is of great importance in many optical applications, ranging from light–matter interaction, imaging and sensing, to optical communication and quantum computation.^1,2^ The conventional methods to control light polarization are through optical elements, such as polarizers and waveplates. However, their bulky nature poses obstacles for the development of compact, efficient, and integratable optical devices. In electrically modulated optical devices, such as liquid crystal displays, polarization control is implemented by liquid crystals (LCs) combined with polarizers. The working LC layers are often several micrometers thick in order to impose sufficient polarization rotation to the propagating light.^3^ Recently, metasurfaces composed of optical antennas in planar layers have become a subject of intense research due to their unprecedented control over light propagation on the subwavelength scale.^4−9^ In particular, metasurfaces allow for efficient phase and polarization manipulation, enabling a plethora of ultrathin optical devices for focusing and lensing,^10−13^ holography,^14−18^ color filtering,^19^ information encryption,^20,21^ among others.^22−28^ Nevertheless, the current endeavors have been mainly devoted to designing and implementing static metasurfaces. There is still plenty of room to develop dynamic metasurfaces,^29,30^ especially through electrical modulation at visible frequencies, to achieve compact, efficient, and ultrathin optical devices, as well as to expand the scope of metasurface optics and related applications. LCs are an ideal material to endow metasurfaces with dynamic functionality due to their stable, reversible, and rapid responses to the external electric fields.^13,14,31,32^

In this Letter, we demonstrate electrically tunable optical metasurfaces for polarization conversion at visible frequencies. The metasurface device contains two main functional components. One is the metasurface itself based on the Pancharatnam–Berry (PB) phase design. Its function is to manipulate the phase of the input linearly polarized (LP) light and split the reflected left-handed circularly polarized (LCP) and right-handed circularly polarized (RCP) light propagating along two different directions. A supercell design comprising plasmonic nanoantennas with opposite orientations in two neighboring rows is adopted to tune the phase delay between the output LCP and RCP light reflected along the same propagating direction. The dynamic tunability is then imposed by the electrical control of the second functional component, the LCs, which cover the metasurface prepatterned with PMMA trenches on the selected nanoantennas. Notably, the utilization of LCs in our device is not for manipulation of polarization but only for modulation of the local refractive index around the defined nanoantennas. Therefore, the working LC layer could be subwavelength thin. This is in contrast to conventional LC-based optical devices, which require thick LCs of several micrometers. Specifically, we experimentally demonstrate electrically tunable optical metasurfaces for polarization rotation, dynamic holography, as well as holographic information generation with independently controlled pixels at visible frequencies.

Figure 1a illustrates the concept of the electrically tunable metasurface device for polarization conversion. The metasuface is encapsulated in a LC cell. An array of gold nanorods with well-defined orientations resides on an indium tin oxide (ITO)-coated SiO_2_/Si substrate, which works as the bottom electrode. Here, Si serves as a back reflector. The gold nanorods are embedded in a thin dielectric polymer (PC403, JCR, green), which serves as a planarization layer and meanwhile helps to eliminate the resonance shifts of the gold nanorods resulting from the refractive index changes of the LCs in response to the applied voltages. The alternating rows are then covered with PMMA (blue) and LCs, respectively. A polyimide alignment layer is rubbed along the direction of the PMMA trenches (*x*-axis). An ITO-coated quartz superstrate works as the top electrode. The incident light is linearly polarized along the *x*-direction. When no voltage is applied, the long-axis of the LC molecules are aligned along the *x*-direction. The reflected LP light will be polarized along the *y*-direction, achieving a 90° polarization rotation compared to that of the incident light. When the applied voltage increases, the orientation of the LCs changes accordingly and so does the refractive index of the LCs (*n*_LC_). The polarization of the reflected light will remain the same as that of the incident light along the *x*-direction. To this end, the polarization of the output LP light can be electrically rotated between 0° and 90° by the metasurface device.

**Figure 1:**
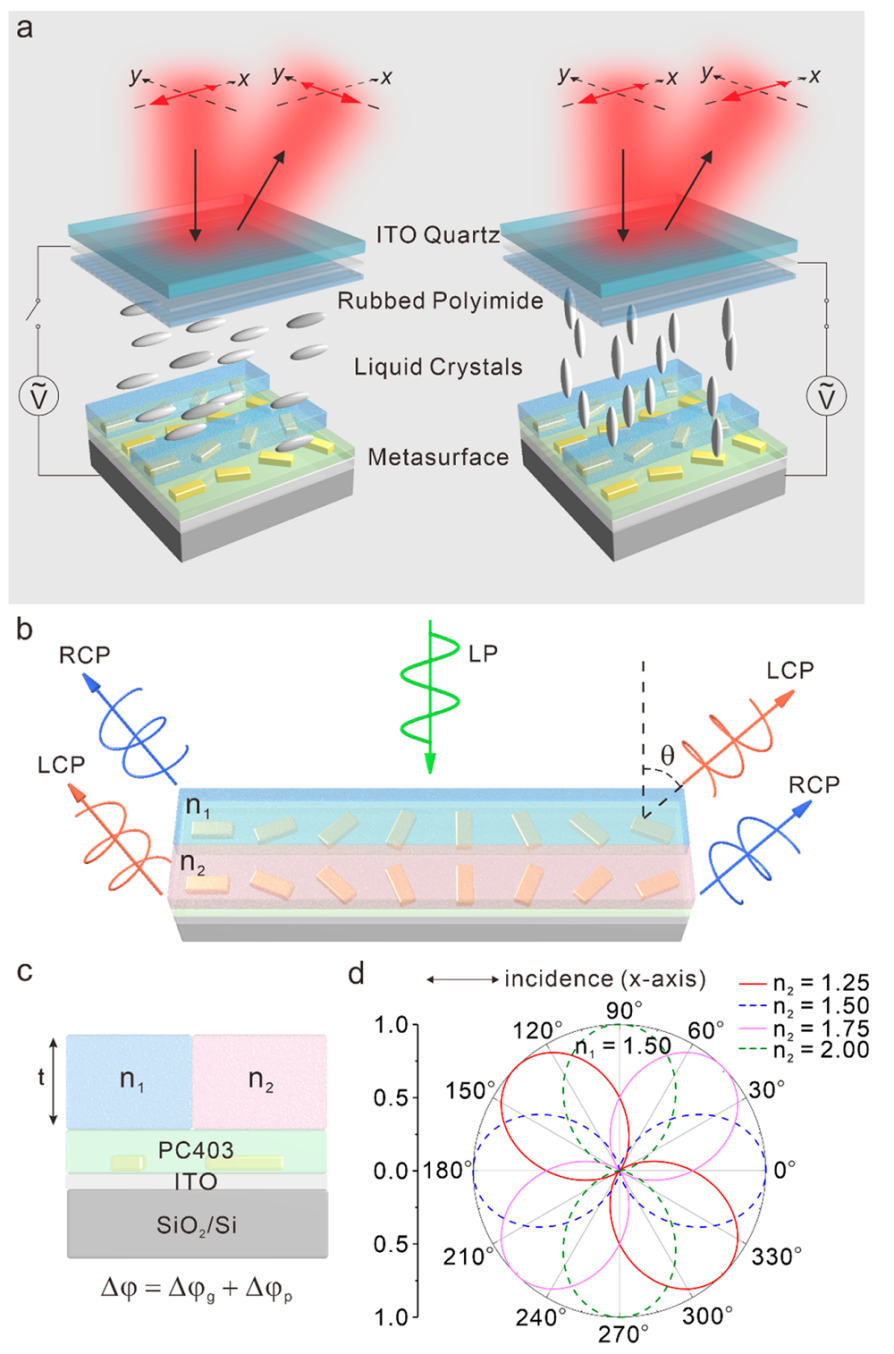
Working principle of the electrically tunable optical metasurface for polarization conversion. (a) An array of gold nanorods embedded in a PC403 (green) layer resides on an ITO-coated SiO_2_/Si substrate, which serves as the bottom electrode. The alternating rows are covered by high-birefringence LCs and PMMA (blue) trenches, respectively. The metasurface is encapsulated in a LC cell. A polyimide alignment layer is rubbed along the direction of the PMMA trenches (*x*-axis). The LC cell is covered by an ITO-coated quartz superstrate as the top electrode. The incident LP light is polarized along the *x*-axis. The LC molecules change their orientations, when the circuit is switched on/off. The polarization of the reflected beam is along the *y*- and *x*-axes, when the circuit is off and on, respectively. (b) Top-view and (c) side-view of the metasurface supercell. Linear polarization generation through superposition of LCP and RCP light under an *x*-polarized light illumination. The PMMA thickness is *t*. The gold nanorods in the neighboring rows are covered by two dielectric materials with refractive indices of *n*_1_ and *n*_2_, respectively. (d) Simulated polarization states of the reflected beam for *n*_2_ = 1.25, 1.5, 1.75, and 2, while *n*_1_ is fixed at 1.5. The rotation angle is defined by the angle formed between the polarization direction of the reflected beam and the *x*-axis.

Figure 1b shows the design schematic of the supercell on the metasurface. Each supercell comprises two rows of gold nanorods embedded in PC403, which are then covered by PMMA (blue, refractive index *n*_1_, thickness *t*) and a dielectric material (pink, refractive index *n*_2_, thickness *t*), respectively. Each gold nanorod has a dimension of 200 nm × 80 nm × 30 nm. They are separated by 300 nm along both the *x*- and *y*-directions. The gold nanorods in the two rows are orientated with angle steps of π/8 and −π/8, respectively, in order to produce the needed 2π phase modulations according to the PB phase. Figure 1c presents the side view of the super cell. The PC403 layer (green) spin-coated on the ITO-coated SiO_2_ (100 nm)/Si substrate has a thickness of 100 nm and a refractive index of 1.5. As illustrated in Figure 1b, when the LP light is normally incident on the metasurface, two reflected beams with opposite helicities, RCP and LCP, are produced by the rows in blue, propagating along two directions −θ and θ, respectively.^33,34^ Here, θ = sin^–1^(λ/*L*_*x*_) = 15.3°, λ is the incident wavelength in free space and *L*_*x*_ is the supercell length. The rows in pink leads to the inverse optical response with the reflected RCP and LCP beams propagating along θ and −θ, respectively.

The reflected LCP (|L⟩) and RCP (|R⟩) beams can be, respectively, described using Jones vectors as
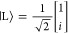

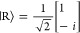
1

The propagation phases introduced by the two covering materials are φ_1_ = *k*_0_*n*_1_*t* and φ_2_ = *k*_0_*n*_2_*t*, respectively. Here, *k*_0_ = 2π/λ is the wavenumber in free space. The superposition of the reflected LCP and RCP waves along the θ direction can be written as
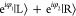
2

The final reflected beam is thus given as
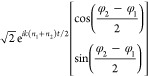
3

It is a LP beam polarized with an angle of  relative to the *x*-axis. The polarization direction depends on λ, *n*_1_, *n*_2_, and *t*. Therefore, the polarization of the output light can be tuned by *n*_2_, when all other parameters are fixed. Meanwhile, along the −θ direction another LP beam is reflected from the metasurface.

To elucidate the underlying mechanism, numerical simulations for the polarization conversion by the measurface device is presented in Figure 1d. In the simulations, the incident LP light at an operating wavelength of λ is polarized along the *x*-direction. The thickness *t* of the two covering materials is set as λ/2. *n*_1_ is fixed at 1.5, while *n*_2_ is varied to different values of 1.25, 1.5, 1.75, and 2 accordingly. As demonstrated by the simulated results in Figure 1d, the polarization direction of the reflected LP light can be tuned by varying *n*_2_. In particular, when *n*_2_ = *n*_1_ = 1.5 (dashed blue), the polarization state of the reflected LP light remain the same as that of the incident light, showing no polarization rotation. A maximum polarization rotation of 90° is achieved, when *n*_2_ = 2 (dashed green) and thus  = . In this regard, the polarization of the reflected LP beam can be arbitrarily rotated between 0° and 90°, relative to that of the incident light through electrical modulation of *n*_2_. Details of the numerical simulations can be found in the Supporting Information.

Next, we experimentally demonstrate the dynamic polarization rotation of the output LP beam by the electrically tunable metasurface device in Figure 2. The LP light (633 nm) polarized along the *x*-axis is normally incident on the metasurface. The two covering materials on the gold nanorod rows are PMMA (*n*_1_ = 1.5) and LCs (*n*_2_ = *n*_LC_), respectively. Here, *n*_LC_ can be electrically tuned from 1.92 to 1.53 by the applied voltage. The PMMA thickness is 410 nm. Details of the structure fabrication and the LC cell construction can be found in the Supporting Information. In order to ensure that the LCs infiltrate well in between the gaps, both the nanorod rows covered by PMMA and LCs are repeated once to double the widths of the PMMA trenches and the gaps, respectively. As shown by the scanning electron microscopy (SEM) image in Figure 2a, the PMMA trenches (gray areas) can be clearly identified. The optical setup to characterize the metasurface device is presented in Figure S1. When the applied voltage *V* is 0 V, *n*_LC_ is equal to 1.92, which yields approximately a phase delay of π between the reflected LCP and RCP beams propagating along the same anomalous angle direction. As a result, the output LP light is polarized along the *y*-axis, achieving a polarization rotation of 90° (see Figure 2b). When the *V* is increased to 20 V, *n*_LC_ is equal to 1.53, which is very close to *n*_PMMA_. The polarization of the output LP light remains approximately the same as that of the input LP light along the *x*-axis (see Figure 2b). In other words, the polarization rotation is 0° in this case. It is noteworthy that here the polarization rotation is mainly due to the presence of the LCs between the PMMA trenches for altering the local refractive index. The LC layer beyond the thickness of the PMMA trenches does not introduce an extra phase delay between the LCP and RCP beams. Therefore, the LCs that contribute to the effective phase modulation is only 410 nm in our metasurface device. This is in contrast to the working principle of the conventional LC-based optical devices. As experimentally demonstrated in Figure 2c, the polarization rotation can be dynamically tuned from 90° to 0° (see gray area in Figure 2c), when the applied voltage is changed from 4 to 20 V. The rotation angle slightly fluctuates, until all the LC molecules in the cell are well aligned at around 4 V. The two insets in Figure 2c depict the measured polarization states for 60° (green) and 30° (red) rotations, respectively. To validate that the observed effect does not result from the rotation of the light polarization imposed by LCs, as a control experiment we have built another LC cell by keeping all the sample settings the same but omitting the gold nanoantennas. As shown in Figure S2 in the Supporting Information, the measured rotation angle is zero and shows no dependence on the applied voltage. To further evaluate the performance of the metasurface device, a polarizer polarized along the *y*-axis is placed on the output path. The output intensity is measured, while the applied voltage is alternatively switched between 0 and 20 V (see Figure 2d). When *V* = 0 V, the reflected beam undergoes a 90° polarization rotation and is thus polarized along the *y*-axis, corresponding to the on state. On the other side, when *V* = 20 V, the reflected beam is polarized along the *x*-axis so that the output intensity is zero after the polarizer, corresponding to the off state. As demonstrated in Figure 2d, the metasurface exhibits excellent reversibility for dynamic polarization rotation, showing no substantial performance degradation up to 100 cycles. The measured switching speed is around 100 ms.

**Figure 2:**
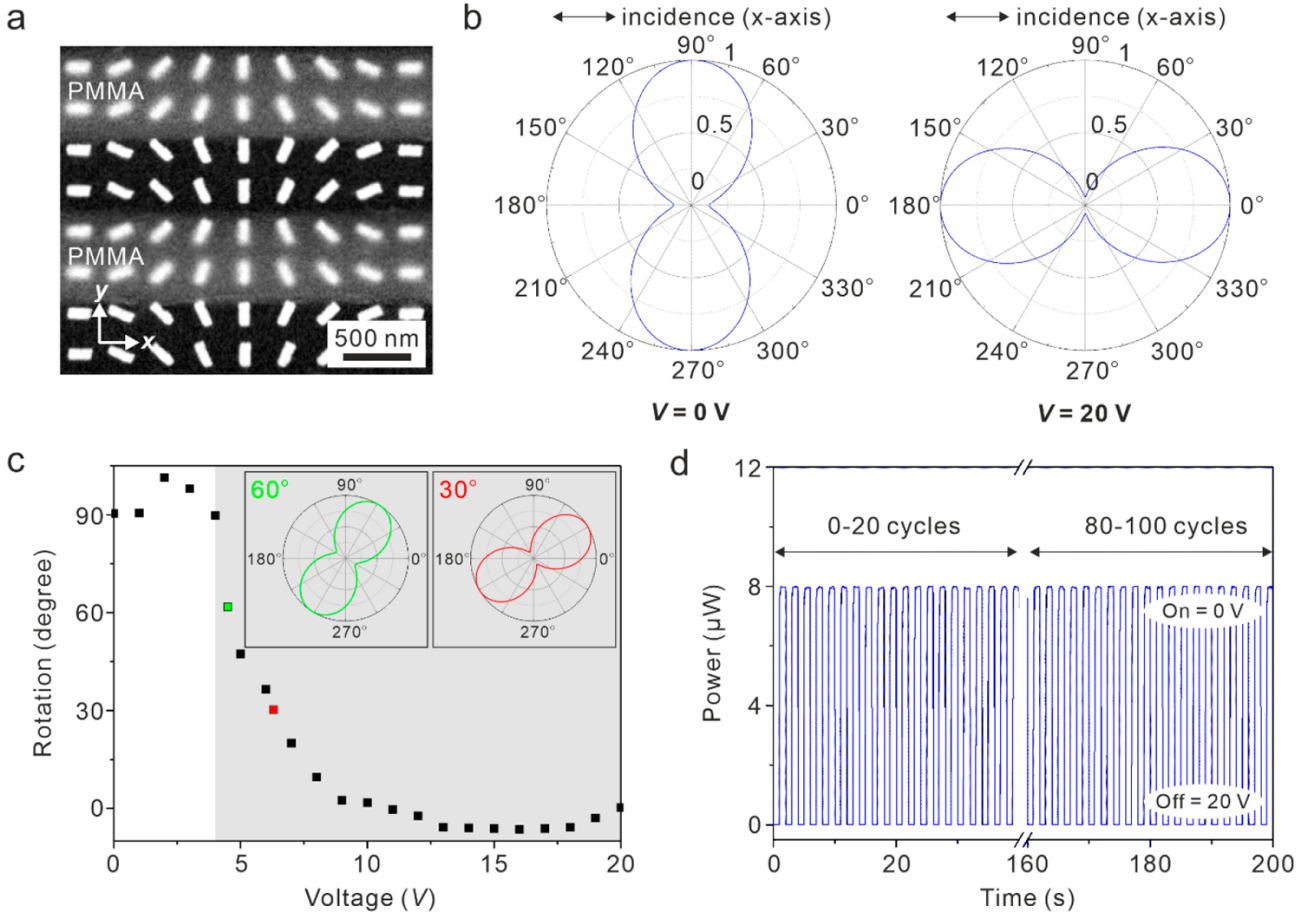
Dynamic polarization control. (a) SEM image of the metasurface sample with PMMA coatings on selective rows. The light gray areas correspond to the coated PMMA trenches. (b) Polarization states of the reflected LP light at *V* = 0 and 20 V, respectively. The incident light at 633 nm is polarized along the *x*-axis. (c) Rotation angle of the linear polarization in dependence on the applied voltage *V*. The polarization states for 60° (green) and 30° (red) rotations are depicted in the insets. (d) Cycling performance of the dynamic metasurface for polarization rotation. The output intensity is detected after the reflected light passes through a polarizer polarized along the *y*-axis.

To demonstrate the versatile application potential of our concept, an electrically tunable metasurface device for dynamic holography is implemented based on the polarization rotation effect (see Figure 3). In this case, the PMMA trench thickness is 470 nm and the single alternating row design is adopted as shown by the SEM image in Figure 3a. The gold nanorods in alternating rows are arranged to generate phase profiles for reconstructing “flower” holographic patterns of left- and right-handed circular polarizations, respectively. Figure 3b depicts the optical setup, in which the incident light at 633 nm is polarized along the *x*-axis and a second polarizer placed on the output path is polarized along the *y*-axis. The hologram intensity in dependence on the applied voltage is measured and shown in Figure 3c. The on and off states of the holographic pattern correspond to *V* = 0 and 50 V, respectively. A higher voltage is utilized here than that in Figure 2, because the single alternating row design is adopted in this case in order to enhance the hologram quality. Meanwhile, it also enlarges the anchoring force of the LCs between the narrower PMMA trenches and therefore requires a higher applied voltage to effectively manipulate the LCs. Through optimizing the metasurface design and developing advanced LC technologies for thin LC layer infiltrations, much lower voltages are needed to operate such LC-based dynamic metasurface devices. Figure 3d presents the captured holographic images at *V* = 0 and 50 V, respectively. The white arrows in the images represent the polarization states of the corresponding holograms. Specifically, the flower pattern occurs at *V* = 0 V along with the polarization rotation to the *y*-axis, whereas it completely vanishes at *V* = 50 V due to the polarization switched to the *x*-axis. The efficiency of the metasurface holograms is around 40%. To demonstrate the broadband nature of our metasurface device, Figure S3 shows the captured holographic images at a wavelength of 520 nm.

**Figure 3:**
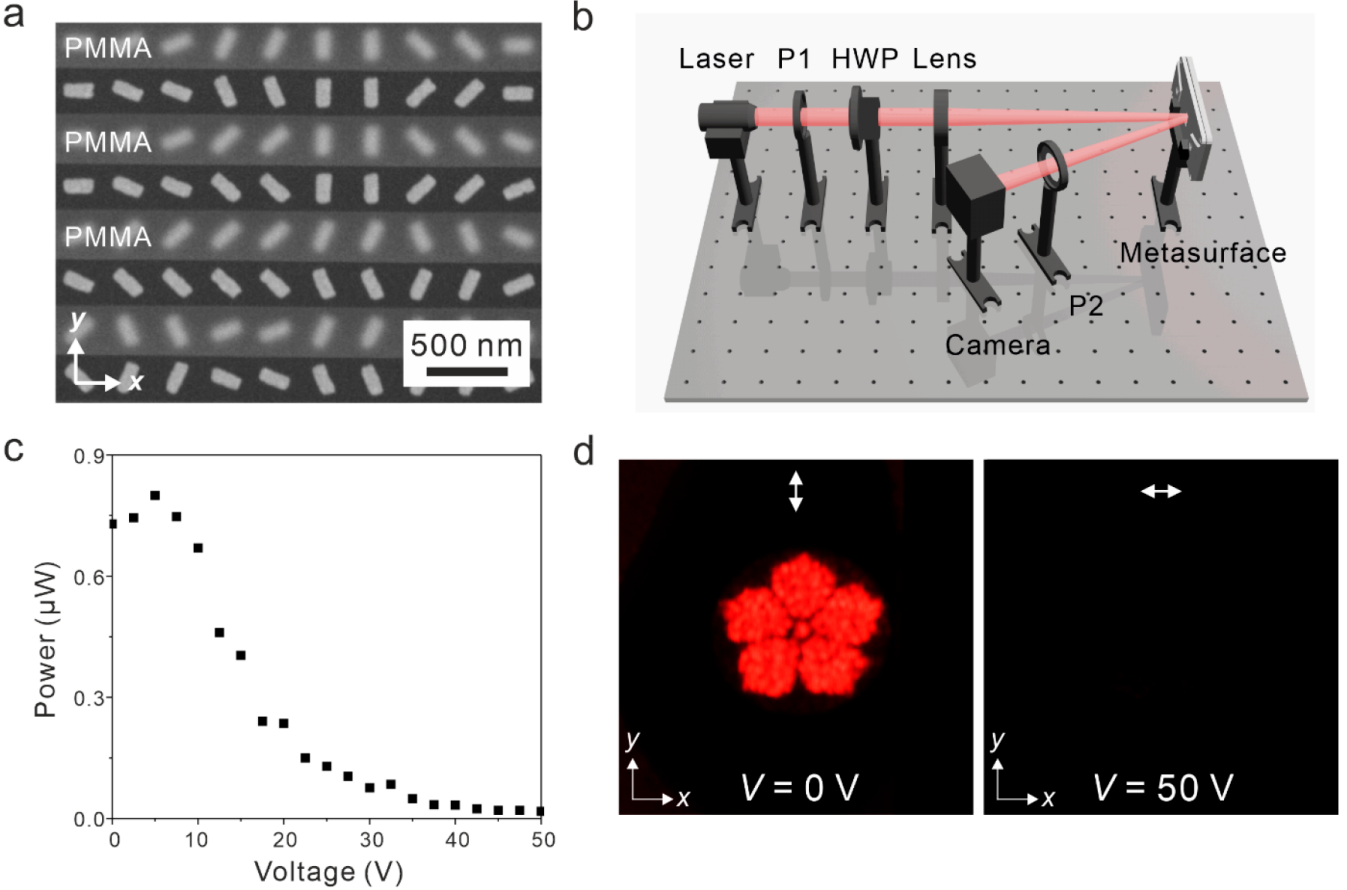
Dynamic holography based on polarization rotation. (a) SEM image of the metasurface sample. (b) Optical setup for characterization of the metasurface holography. P1 and P2 represent polarizers. HWP represents a half wave plate. (c) Detected light power in dependence on the applied voltage. (d) Holographic patterns at the on and off states, corresponding to *V* = 0 or 50 V, respectively. White arrows indicate the polarization directions of the holograms.

Taking a step further to expand the capability of our concept, independent control over multiple metasurface pixels for holographic information generation is demonstrated. As shown by the SEM image of the device in Figure 4a, two metasurface areas, called pixels (M1 and M2), are controlled by two independent ITO electrodes 1 and 2, respectively. M1 and M2 are utilized to generate and switch on/off holographic patterns of “0” and “1”, respectively, as illustrated in the inset images in Figure 4a. The experimental results are shown in Figure 4b. The holographic pattern “0” can be switched on and off at *V*_1_ = 0 and 50 V, respectively. Meanwhile, the holographic pattern “1” can be switched on and off at *V*_1_ = 0 and 50 V, respectively. The corresponding polarization states of the holographic patterns are indicated using white arrows in Figure 4b. In this regard, four states containing different holographic information can be reconstructed by the electrically tunable metasurface device via independent control over multiple pixels.

**Figure 4:**
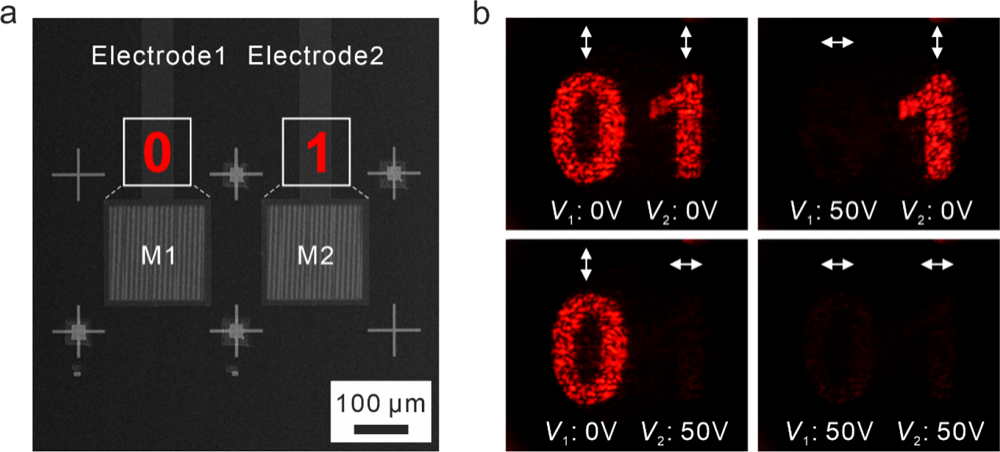
Holographic information generation with independently controlled metasurface pixels. (a) SEM image of the sample. Two addressable metasurface pixels (M1 and M2) are controlled via two independent ITO electrodes. Insets: schematics of the reconstructed holographic patterns from M1 and M2. (b) Holographic information at four different states is generated by electrically controlling the two independent metasurface pixels.

In conclusion, we have demonstrated electrically tunable metasurfaces for polarization conversion at visible frequencies, which enable fast and reversible polarization rotation, dynamic holography, and holographic information generation with independently controlled multiple pixels. Compared to the conventional LC-based devices, which rely on micrometer thick LC layers to enable polarization rotation of light, our metasurface devices combine both the tuning capabilities of the geometric and propagation phases for polarization conversion. The LCs, whose refractive index is electrically tunable, work as a surrounding medium for the predefined nanoantennas. Our design scheme can also be extended and employed to realize dynamic polarization conversion in a transmission mode based on a transparent substrate. Our work will shed light on novel design principles for the realization of compact, efficient, and integratable metasurface devices with versatile dynamic functionalities at visible frequencies.

## Supporting Information Available

Numerical simulations and sample fabrication details, optical setup (Figure S1), and additional experiments results (Figures S2 and S3) (PDF)
