# Supplementary material for: Electrically Tunable Optical Metasurfaces for Dynamic Polarization Conversion
Source: Nano Lett. 2021 Jul 21;21(15):6690–5. doi: 10.1021/acs.nanolett.1c02318 (PMC8361430; doi:10.1021/acs.nanolett.1c02318)
Supplement: Supplementary file 1 — nl1c02318_si_001.pdf [file nl1c02318_si_001.pdf]

Electrically-tunable optical metasurfaces for dynamic polarization conversion

*Ping Yu[1#], Jianxiong Li[1#], and Na Liu[1,2,]**

[1]Max Planck Institute for Solid State Research, Heisenbergstrasse 1, 70569 Stuttgart, Germany.
[2]2nd Physics Institute, University of Stuttgart, Pfaffenwaldring 57, 70569 Stuttgart, Germany.

#These authors contributed equally to this work.

*Email: na.liu@pi2.uni-stuttgart.de

Numerical simulations, sample fabrication details, optical setup, and additional experiments results

Numerical simulations

Numerical Simulations in Figure 1d were carried out using commercial software COMSOL Multiphysics based on a finite element method. Periodic boundary conditions were utilized for the super unit cell along the x- and y-directions. A waveguide port boundary condition was used as the excitation source that was normally incident onto the structure. The incident linearly polarized light was defined with an electric field (Ex = 1, Ey = 0, Ez = 0). Perfectly matched layers were placed at the top and bottom of the simulation domain to completely absorb the waves leaving the domain. The mesh size was fixed as 10 nm in the simulation domain. The substrate was included in the simulations. The refractive index of SiO_2 was taken as 1.5. The dielectric constant of gold was taken from Palik.[1]

Structure fabrication

The samples were fabricated using multi-step electron beam lithography (EBL). First, a structural layer composed of the electrode patterns and alignment markers were defined in ARN resist using EBL on an ITO-coated SiO_2 (100nm)/Si substrate. The ITO electrode patterns and markers were then achieved through ITO etching. Subsequently, the substrate was coated with a double PMMA layer. Computer-controlled alignment using the markers was carried out to define a second structural layer composed of the gold nanorods. A 2 nm Cr and 30 nm gold were deposited on the substrate using a thermal evaporator followed by a lift-off procedure. Next, a 100 nm PC403 (JCR, Japan) layer was coated on the substrate. A prebaking process was first carried out to remove the solvent from the polymer by increasing the baking temperature from 90 °C to 180 °C. A longer baking process at 180 °C for 30 min was then applied. Finally, PMMA trenches were fabricated using EBL.

LC cell construction

A rubbed-polyimide ITO-coated glass slide was adhered to the metasurface sample using ultraviolet-cured glue (NOA 81) containing 5-μm glass spacer beads. After the glue was cured using ultraviolet light, the cell was heated to 120 °C and infiltrated with LCs (LCM1107). Phase formation of the LCs took place after cooling to room temperature.

Optical setups

The light beam was generated from a laser diode source (633 nm). A linear polarizer (LP) and a half waveplate (HWP) were employed to obtain linearly polarized light. A lens is utilized to reshape the light beam to a similar size as the sample area in order to avoid undesired reflected light. The polarization state of the reflected anomalous beam was detected by a polarimeter, as shown in Figure S1.

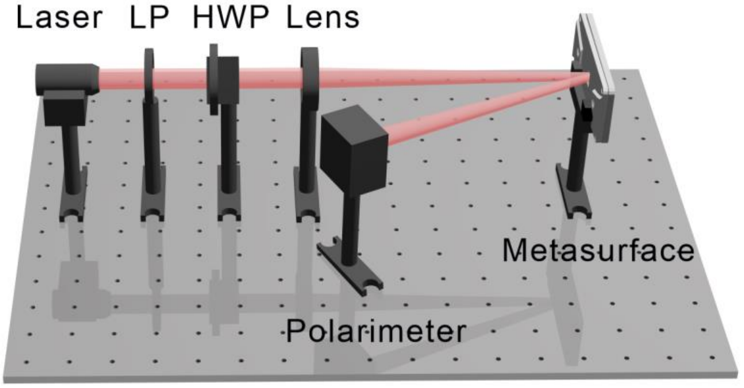

Figure S1. Schematic of the optical setup for the polarization state detection. LP and HWP represent linear polarizer and half waveplate, respectively.

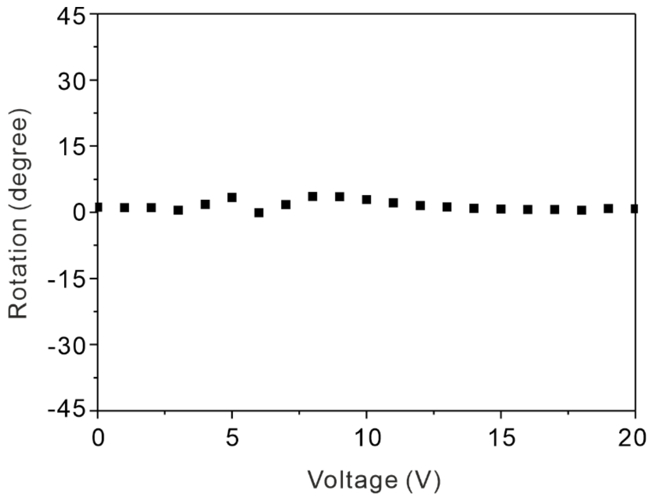

Figure S2. Control experiment. Rotation angle of the linear polarization in dependence on the applied voltage *V*, when the gold antennas are omitted in the LC cell, while all other sample settings are kept the same.

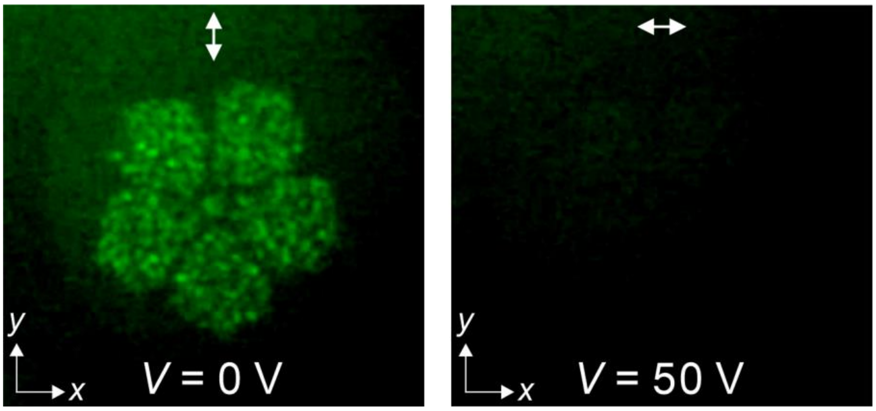

Figure S3. Holographic patterns at the on and off states, corresponding to $V = 0$ V or 50 V, respectively, at an operating wavelength of 520 nm. White arrows indicate the polarization directions of the holograms.

References

1. Palik E. D. *Handbook of Optical Constants of Solids,* Academic Press, **1998**.
